# Evaluating the Influence of Water Scarcity on the Host Response of Garlic to the Stem and Bulb Nematode *Ditylenchus dipsaci*

**DOI:** 10.3390/plants12223845

**Published:** 2023-11-14

**Authors:** Carmen Lorenzo, Fabio Ramos, Andrés Casado, Ana-María Gálvez, Soledad Sanz-Alférez, Gloria Nombela

**Affiliations:** 1 Institute for Agricultural Sciences (ICA), Spanish National Research Council (CSIC), Serrano 115 Dpdo., 28006 Madrid, Spain; carmenlr@ica.csic.es (C.L.); fabio423@gmail.com (F.R.); andrescasadobecerra97@gmail.com (A.C.); agalvez@ias.csic.es (A.-M.G.); 2 Departament of Biology, Universidad Autónoma de Madrid (UAM), Campus de Cantoblanco, 28049 Madrid, Spain; soledad.sanz@uam.es

**Keywords:** *Allium sativum*, chlorophyll concentration, *Ditylenchus dipsaci*, drought, garlic, physiological changes, plant parasitic nematodes, proline accumulation, water stress

## Abstract

*Ditylenchus dipsaci* is a plant-parasitic nematode with a great economic impact on bulbous crops, including garlic (*Allium sativum* L.), and is distributed worldwide, particularly in the Mediterranean region. Traditionally, garlic was a rainfed crop in Spain, but irrigated areas have increased during the last few decades. However, the expected climatic conditions, with longer and more intense droughts, will make it necessary to reduce the water supply to garlic crops. This poses the urgent need to select garlic cultivars more tolerant to water scarcity and that are also more resistant to plant pathogenic organisms. The aim of this work was to analyze the influence of water stress on the host response of garlic plants to *D. dipsaci*. The specific objectives were to evaluate the level of nematode infestation in plants from four garlic genotypes treated with a reduced irrigation regime and compare them with those of control plants not subjected to water stress. The observed results were correlated with changes in the bulb and root development, as well as in the physiological parameters (total chlorophyll concentration and proline accumulation). The effects were different depending on whether the plants were subjected to water stress before or after nematode inoculation, as well as whether the water stress was continuous or discontinuous. Garlic inter-cultivar variability also affected the obtained results.

## 1. Introduction

Garlic (*Allium sativum* L.) is a bulbous plant belonging to the *Amaryllidaceae* family, native to central Asia, with secondary diversification zones in China and the Mediterranean region [1]. It is a globally distributed crop of huge importance in several Asian and Mediterranean countries, due to its gastronomic properties [2], and having been used since ancient times as a traditional medicine [3,4]. The total area dedicated to this crop has increased by around 250% over the last 50 years, with more than 1.6 million ha in 2021, and the total world production has increased 8.9 times, surpassing 28 million tons in 2021 [5]. Spain is considered one of the main garlic producers in Europe, with a total production of 315,720 tons [5], with Castilla–La Mancha being the region with the most abundant garlic production in the country [6]. Historically, garlic has been a rain-fed crop in Spain [7]. Despite this, the competitive agronomic market has made it necessary to increase the irrigated garlic crop area, in order to obtain greater production. Nowadays, more than 90% of garlic crops in Spain are irrigated [6]. Although irrigation has increased the productivity of garlic crops [8], it has also caused conditions that favor the development of certain pests [9].

Garlic plants can be attacked, throughout their different development stages, by different pathogenic organisms, such as fungi, viruses, bacteria, and nematodes [10]. These attacks can affect garlic crop production and quality [11], causing important economic losses as infected crops are unmarketable [12]. Among these organisms, *Ditylenchus dipsaci* (Kühn) Filipjev, also known as the “stem and bulb nematode” [13], is a strict obligate migratory endoparasite [14,15] of more than 500 plant species [16,17,18] in over 40 plant families [13]. The principal hosts are *Allium cepa*, *A. sativum*, *Avena sativa*, *Fragaria* spp., *Medicago sativa*, *Narcissus* spp., *Tulipa* spp., and *Vicia faba* [15]. *D. dipsaci* is composed of numerous biological races, which are generally not morphologically distinguishable [16]. *D. dipsaci* is considered one of the most damaging parasitic nematode species in bulbous plants [19] and is one of the main pests in garlic crops [20]. *D. dipsaci* is probably native to Europe [17] and it has a worldwide distribution, principally in temperate regions [12,21]. At the global level, annual losses to garlic crops caused by *D. dipsaci* are estimated to be around 60–80% of the yield in heavily infested cases [13]. This nematode is very widespread in Spain [22], and garlic crop losses caused by *D. dipsaci* in the Castilla–La Mancha region are estimated to be around 40–60% of the total harvest [23].

*D. dipsaci* is infective from the second juvenile stage (J2) to the adult stage, J4 being the principal infective stage [15], and feeding primarily on parenchyma plant tissue [16]. In garlic, it produces yellowing and leaf death [24], discoloration [16], and swelling [10] of the bulb tissues, and rotting of the basal plate [14]. When the infected plant dies, *D. dipsaci* remains in the soil in an anhydrobiosis state that can last many years [16]. This allows the *D. dipsaci* population to persist in infested soils [15,16,25]. This ability of *D. dipsaci* to survive under drought conditions is an important factor to be taken into account in a climate change context, which is having a negative impact on agricultural systems in the short term, causing important instability in crop yields over seasons and, as a consequence, economic losses [26].

Longer and more frequent droughts are expected in the Mediterranean region [27], with up to a 20% precipitation decrease predicted for 2080–2099, compared with 1980–1999 data [28]. Drought stress generates physiological changes in higher plants, including the loss of turgor pressure, osmotic balance, and reduced leaf water potential [29], which leads to the closing of the stomata and impaired growth [30], as well as the production of reactive oxygen species (ROS), which subsequently damage the photosynthetic machinery [31]. Lower plant biomass then results from diminished photosynthesis and cellular respiration, and less efficient uptake of ions, sugars, and other nutrients occurs, including the translocation of photosynthetic products [32,33]. In general, plant cells make osmotic adjustments by increasing the accumulation of osmolyte solutes [34] to counteract lower water potential. Some of the key osmolytes in plants include quaternary ammonium compounds (e.g., glycine betaine and choline) and proline [35]. Proline is critical for protein synthesis and plant development; free proline accumulation in water-stressed leaves has been speculated to constitute an attribute of drought resistance or drought hardiness.

In addition to drought and other abiotic factors, biotic stresses such as an attack by pathogenic organisms can affect plant growth and yield. Plants have developed a complex morphological, metabolic, and molecular response system to prevent and/or tolerate stress damage and survive [36]. The response by plants to simultaneous biotic and abiotic stresses is distinct from individual stresses, and is not merely additive [37,38]. In this regard, the combination of a water deficit and plant-parasitic nematodes is a realistic threat under field conditions and could drastically impact crop productivity. Drought stress itself generates many physiological changes in higher plants, as detailed above. However, nematode interaction can intensify or neutralize the effects of water stress on plants, as root parasitism greatly influences the plant–water relations [39].

Previous works have studied how water stress affects the host response in different crops to plant-parasitic nematodes, such as *Meloidogyne* [40,41,42,43], *Heterodera* [44], and *Pratylenchus* [41,45]. These studies have shown how water stress is, in most cases, a relevant factor in reducing the general development of plants, also negatively affecting nematode reproduction. The main goal of the present study is to evaluate, for the first time, the impact of water stress on the host response of garlic to the attack by the nematode *D. dipsaci*. For this purpose, the levels of nematode infection in plants from four garlic varieties subjected to a reduced irrigation regime were compared to those of similar plants with more abundant irrigation. The influence of the time points at which the plants underwent water stress was analyzed, as well as whether that stress was continuous or discontinuous. The effect of water stress on bulb and root development in plants from the same garlic varieties was also evaluated. In addition, the obtained results were correlated with changes in the physiological parameters (total chlorophyll concentration and proline accumulation) due to water stress and nematode infestation.

## 2. Results

### 2.1. Effect of Water Stress on Nematode Infestation

When the plants were inoculated with *D. dipsaci* after 4 weeks under a reduced irrigation regime and this water stress condition was maintained until the end of the assay, the final number of nematodes per plant was lower than that of the control plants, with statistically significant differences in all four garlic cultivars studied (Table 1).

When the water stress treatment was applied 2 weeks after nematode inoculation or to naturally infested plants (Table 2), the results were different to those previously shown in Table 1 (water stress prior to nematode inoculation): no significant differences were found between the C and S treatments in the GA, FC, and VS cultivars. In the case of MP, the S plants had significantly more nematodes than the C plants.

### 2.2. Effect of Water Stress on Physiological Response of Garlic

**Figure 1:**
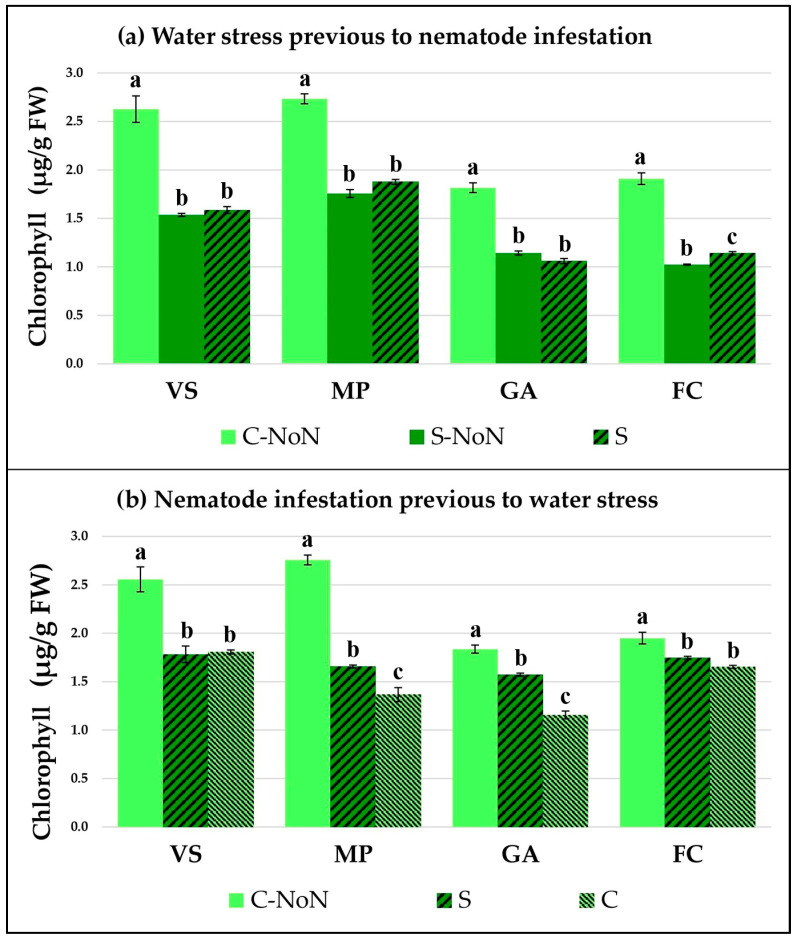
Total chlorophyll concentration in four garlic cultivars: Violeta Spring (VS), Morado de las Pedroñeras (MP), Gardacho (GA), and Fino de Chinchón (FC). (**a**) Plants subject to an initial water stress treatment (S) or without water stress (C or control) and later infestation by *D. dipsaci*. (**b**) Plants with an initial infestation by *D. dipsaci* and later water stress (S) or the control (C) treatment. NoN: plants with no nematodes. Within the same garlic cultivar, different letters (a, b, or c) represent significant differences between the water treatments, according to the Kruskal–Wallis test.

**Figure 2:**
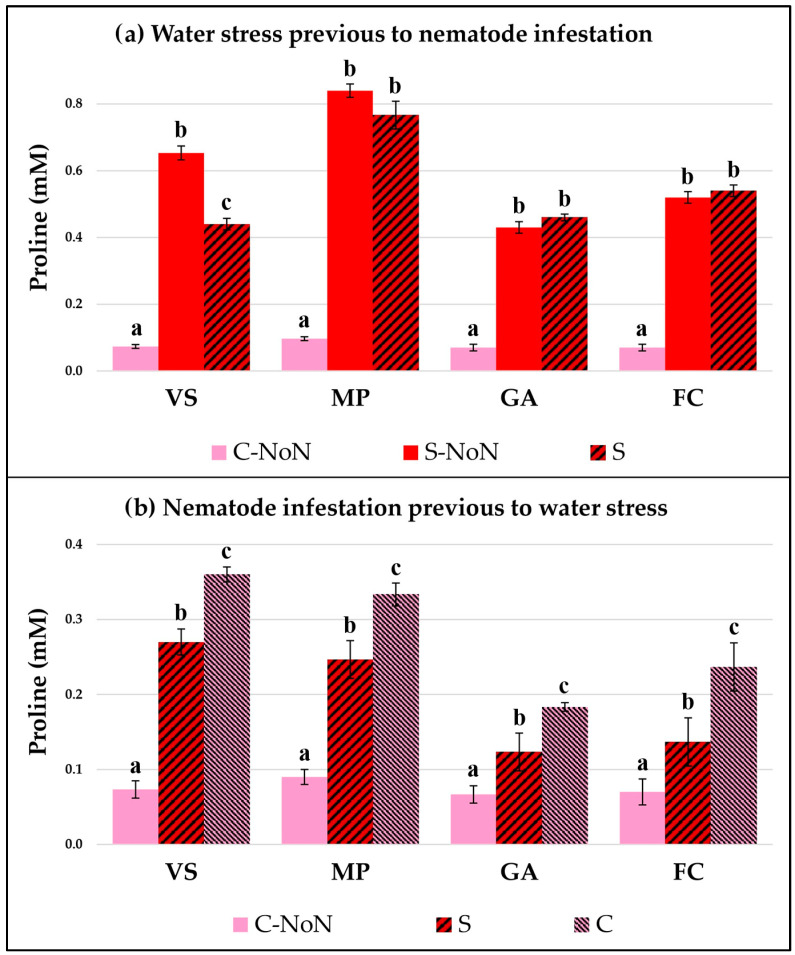
Proline accumulation in four garlic cultivars: Violeta Spring (VS), Morado de las Pedroñeras (MP), Gardacho (GA), and Fino de Chinchón (FC). (**a**) Plants subject to an initial water stress treatment (S) or without water stress (C or control) and later infestation by *D. dipsaci.* (**b**) Plants with an initial infestation by *D. dipsaci* and later water stress (S) or the control (C) treatment. NoN: plants with no nematodes. Within the same garlic cultivar, different letters (a, b, or c) represent significant differences between the water treatments, according to the Kruskal–Wallis test.

The plant response by each garlic cultivar was studied by checking two physiological parameters: the total chlorophyll concentration (Figure 1) and the proline accumulation (Figure 2). Both of them indicate how the plant metabolic and stress responses are affected by the different treatments in our experimental conditions.

When water stress occurred prior to the nematode infestation (Figure 1a and Figure 2a), both parameters were modified in all the tested cultivars. In general, the chlorophyll content was reduced and the proline concentration was increased due to water stress compared with the untreated and non-infested plants (C-NoN). The data showed higher augmentation of proline in MP after water stress, which was maintained at a high level with the nematode inoculation. On the contrary, a negative effect was observed for water stress on the chlorophyll concentration in all cultivars and an important reduction was detected, indicating that the garlic metabolism was affected during both water stress and later nematode infestation.

Prior to the application of water stress to the garlic plants, nematode infestation by itself produces changes in the physiological parameters, reflected by changes in the chlorophyll concentration and proline accumulation in the leaves (Figure 1b and Figure 2b). The nematode feeding activity reduces the available water in the plant and induces the production of proline, as well as modifying the plant’s basic metabolism, represented by the chlorophyll concentration. However, the stress response in all the garlic varieties tested reflected very similar patterns after the subsequent reduction of irrigation, which was an increase in proline in parallel with a reduction in chlorophyll in relation to the control (C). However, the VS and MP presented a great rise in proline concentration, compared to the GA and FC garlic varieties.

### 2.3. Effect of Discontinuous Water Stress on Nematode Infestation

**Figure 3:**
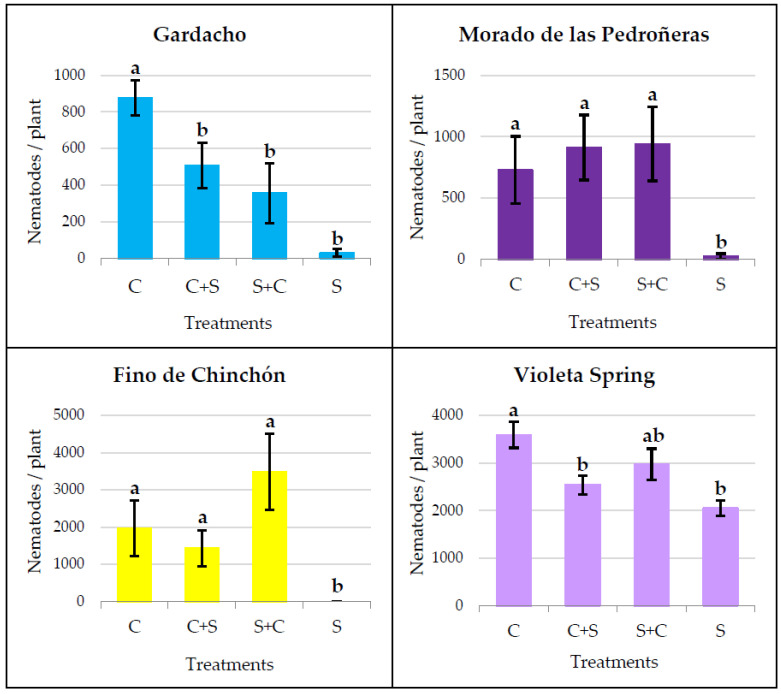
Number of nematodes per plant, obtained when the water treatments were continuously or discontinuously applied. C: continuous control, C+S: control prior to stress, S+C: stress prior to control, S: continuous stress. Within the same garlic cultivar, different letters (a or b) represent significant differences between the water treatments, according to the Kruskal–Wallis (for GA, MP, and FC) or Tukey’s HSD post hoc test (for VS).

The number of nematodes obtained when stressed plants were switched to a normal watering regimen, 4 weeks after nematode inoculation and 9 weeks after starting the water stress (S+C) or vice versa (C+S), were compared to those from other plants where the usual water treatment (C or S) was continued until the end of the assay (Figure 3).

Statistically significant differences among the water treatments were detected in all four garlic cultivars, according to the Kruskal–Wallis (H value) or the ANOVA (F value) test: GA (H = 16.217 *p* < 0.001), MP (H = 10.027 *p* = 0.018), FC (H = 20.269 *p* < 0.001), and VS (F = 6.547 *p* < 0.001).

The significant differences between the continuous C and S treatments were maintained in all garlic cultivars and no significant differences were observed between the C+S and S+C treatments. However, the results were highly variable in the case of the S+C and C+S plants, depending on each garlic cultivar. So, the cultivars MP and FC showed a similar pattern, with a certain increase in the number of nematodes in the S+C plants with respect to the C and C+S; although, these differences were not statistically significant. In the case of the GA and VS, the final infestation levels for the S+C and C+S were not significantly different from that of the S plants.

### 2.4. Effect of Water Stress on Bulb and Root Development

When the root and bulb weights were compared among the different water treatments, statistically significant differences were detected in all four garlic cultivars, according to the ANOVA (F value) test: GA (F = 8.364 *p* < 0.001), MP (F = 6.200 *p* = 0.003), FC (F = 54.628 *p* < 0.001), and VS (F = 19.369 *p* < 0.001).

**Figure 4:**
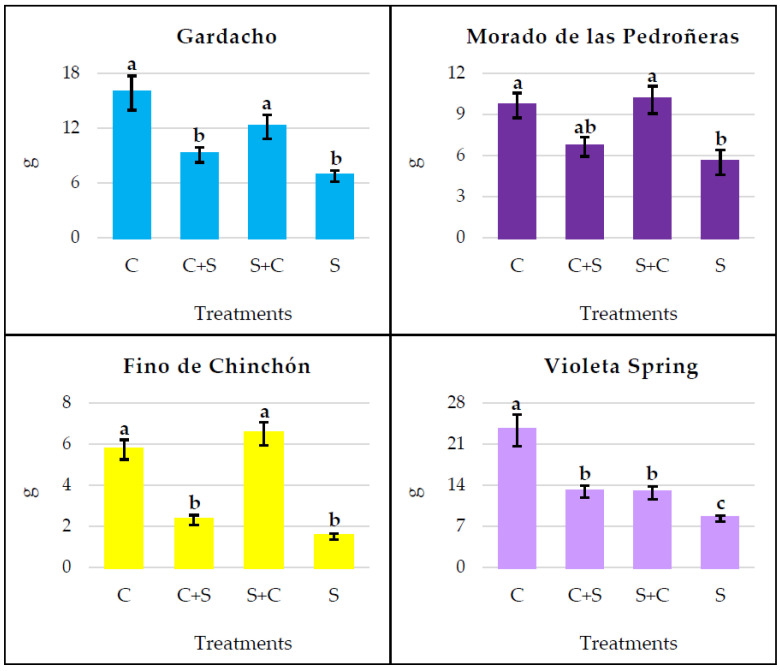
Weight of bulb and roots per plant, obtained when the water treatments were continuously or discontinuously applied. C: continuous control, C+S: control prior to stress, S+C: stress prior to control, S: continuous stress. Within the same garlic cultivar, different letters (a, b, or c) represent significant differences between the water treatments, according to Tukey’s HSD post hoc test.

The plants under continuous water stress (S) showed a significantly lower weight than the control plants (C) in all four garlic cultivars (Figure 4).

The discontinuous application of both water treatments caused intermediate values for the bulb and root weight in the Gardacho and Violeta Spring cultivars. However, in the Morado de las Pedroñeras and Fino de Chinchón, the S+C plants had similar weights to the C plants, and significantly greater weights than those subjected to continuous stress (S). Overall, the weight values for the C+S plants were slightly higher than those for continuous stress; although, this difference was statistically significant only in the case of Violeta Spring.

## 3. Discussion

The obtained results from this work consistently show the importance of the timing of the water stress in the host response of garlic plants to *D. dipsaci*, since the influence of this abiotic factor diverged significantly if the plants suffered water stress before or after nematode infestation. A reduction in the amount of water used for irrigation of garlic plants prior to *D. dipsaci* inoculation leads to a significant decrease in the final nematode infection levels compared to plants watered more abundantly. This scenario occurred in all four tested garlic cultivars (Gardacho, Morado de las Pedroñeras, Fino de Chinchón, and Violeta Spring), and it is probably because the lack of water hinders the nematode mobility in the soil and their penetration of the root tissues. Similarly, water stress prior to nematode infestation affected the ability of *Meloidogyne incognita* to mount an efficient parasitism in tomato plants, as a lower nematode penetration rate was observed leading to a drastic reduction in the number of galls [46]. Coincident with this, the introduction of soil moisture decreasing practices, such as summer fallow, is often used prior to wheat sowing to reduce the incidence of *Heterodera avenae* [47]. Decreasing the penetration success of *D. dipsaci* in garlic plants due to drought is relevant because this is a species with a fast reproductive rate [16], and the swift expansion of the *D. dipsaci* population can lead to extensive agricultural losses, even if the initial population density in the soil is minimal [12].

In contrast, when previously infested garlic plants were subjected to reduced irrigation, a significant increase in *D. dipsaci* populations was observed in the Morado de las Pedroñeras, compared to plants of the same cultivar under control conditions. For the other three cultivars tested, the final number of nematodes in the stressed plants was also greater or similar than those in the control plants, but the differences were not statistically significant. The effects caused by water stress in garlic can be compared with previous studies on other nematodes affecting different crops. For instance, it is well known that the damage caused by cereal cyst nematodes (CCNs) can be enormous when they occur in areas subject to water stress [48]. However, the increase in the mean cyst density of *Heterodera sacchari* in rice plants from the initial density was greater under continuous irrigation than under drought conditions [44]. These examples reinforce the importance of taking into account the relative timing of the reduction in water supply in each case before assessing the impact of water stress on the plant’s host response to a nematode attack. Overall, the results in the present work indicate that a shortage of irrigation water supply in garlic cropping can be particularly useful to manage *D. dipsaci* populations, if it is implemented prior to garlic planting, avoiding overwatering.

The results from the present work were similar regardless of whether they were obtained in a climatic chamber or in a shade house, which provided environmental conditions closer to those normally found in garlic cultivation. This supports the validity and usability of this type of bioassay for application and decision making in a variety of environments, including open field crops. Regarding another aspect of the methodologies followed in this work, direct nematode counting is very reliable, but quite laborious and time consuming. Technological advances are allowing the emergence of more sophisticated computerized methods, which can also provide high throughput counts without being time consuming. For instance, a high-resolution scanner can be used for taking images of the nematode suspension, together with deep learning algorithms to identify and count the nematode eggs. Another approach is a lensless imaging setup to take real-time, holographic videos of the processed sample passing through a microfluidic flow chip, which is analyzed with a custom software program [49].

The metabolism and growth were affected by both stresses applied to the garlic plants in our experiments, water stress and infestation by *D. dipsaci*. The reduction in the total chlorophyll detected indicates that the plant metabolism and the photosynthetic activity are negatively altered [30,32,33]. This could be used as an indicator to identify plants affected by these stresses. Furthermore, the plant’s response to water stress and nematode inoculation was induced by an increment in the proline osmoregulator, reaching an equilibrium with the soil water potential to maintain the nutrient solution transportation [35]. Other metabolic parameters could be used to check the plant responses to our treatments, such as the sugar content, protein concentration, ROS accumulation, and phytohormone alteration, among others. Since drought stress decreases the water content and leaf water potential, this leads to the closing of the stomata and impaired growth [30]. On the other hand, lower plant biomass results from diminished photosynthesis and cellular respiration, and less efficient uptake of ions, sugar, and other nutrients occurs, including the translocation of photosynthetic products [32,33]. Another effect of drought stress is the production of reduced components from the photosynthetic electron transport chain leading to the production of reactive oxygen species (ROS), which subsequently damage the photosynthetic machinery [31]. It is relevant to mention that the four cultivars behave differently. The proline concentrations reached in the VS and MP cultivars caused by water stress were higher than the levels of the GA and FC, suggesting a better tolerance to drought. Also, the nematode infestation increases this metabolite to a very similar level, since *D. dipsaci* extracts water and metabolites from the garlic roots and the plant responds to this water depletion. The proline induction in garlic caused by *D. dipsaci* infestation before water stress treatment reached a similar level to that of water stress prior to nematode infestation, highlighting that the garlic plants responded in the same way to both stress factors. Finally, we observed that the chlorophyll concentration decreased similarly in the four cultivars studied.

When water stress occurred discontinuously, it was not possible to find a clear pattern except that, in these assays, the previously demonstrated difference between the continuous control and stress treatments, applied prior to nematode inoculation, was confirmed for all the garlic cultivars tested. Other than this, no significant differences were observed between the two groups of plants where water stress was applied discontinuously (C+S and S+C). This result was also consistent for all four tested cultivars. Moreover, in the Gardacho or Violeta Spring cultivars under discontinuous treatments (C+S and S+C), the final number of nematodes was not significantly different from those in the plants continuously subjected to water stress (S). For the same cultivars, when the water shortage followed a first period of normal irrigation (C+S), the infestation level was significantly lower than that of the control plants. On the other hand, the Morado de las Pedroñeras and Fino de Chinchón cultivars showed a similar pattern to each other, with a certain increase in the number of nematodes when the water stress was transient, followed by a period of abundant watering (S+C), with respect to the rest of the treatments. However, the differences with the C and C+S plants were not statistically significant. Due to the variability in these results, it is not possible to draw an overall conclusion, but it can be confirmed that the discontinuous application of water stress differentially affects the host response of garlic cultivars to infestation by *D. dipsaci*. This could, perhaps, be due to some pre-existing differences among the garlic cultivars in their basal host response to a nematode attack.

The differential results obtained in garlic in the present work show the influence of the moment at which the plant begins to suffer the consequences of water stress, as well as the importance of inter-cultivar variability.

As shown in previous studies, irrigation is a determining factor for bulb parameters, including the size [50,51], weight, and number of cloves [52], which all are smaller when faced with a water deficit. In the present work, it has been confirmed that more abundant irrigation favors the plant metabolism and photosynthetic activity, with greater growth in both the garlic bulbs and roots. In the absence of water stress, the plants from all four cultivars analyzed exhibited a significantly higher weight. Consequently, when irrigation is reduced, the plant response to water stress is induced and the size of the garlic bulb and roots are smaller. This is in agreement with a number of previous studies and, particularly, a recent work by the collaborators in this project [53], where similar results were obtained for the bulb and root weights for the same garlic varieties studied here (GA, MP, FC, and VS). As demonstrated by other authors [54], seasons with lower rainfall negatively affect bulb size at harvest. This again confirms that the obtained results in the present work, under controlled conditions, are applicable to crops in open field conditions.

Considering the limitations to water use for overall agricultural yields [55], and drought being the most important environmental stress factor for crops [56], the results from the present work lead us to consider that garlic cultivation in arid areas should preferably follow the traditional rainfed system in the near future, which will help to reduce the risk of yield losses due to plant-parasitic nematodes, such as *D. dipsaci*. Moreover, genetic erosion is steadily increasing, due to the substitution of local and traditional varieties and cultivars in garlic cultivation by commercial, higher yielding, and economically more profitable ones. This scenario diminishes crop genetic variability and their potential to adapt to forthcoming environmental changes, as well as to emerging pests and pathogens, making farming systems less resilient and missing potential sources of crop enhancement [57,58]. Hence, under water scarcity circumstances, it will be necessary to select garlic varieties that exhibit higher resistance, not only to drought, but also to nematodes and other plant-pathogenic organisms, to minimize economic losses.

## 4. Materials and Methods

### 4.1. Plant Material and Garlic Germination

Four garlic cultivars were tested: Gardacho (GA), Morado de las Pedroñeras (MP), Fino de Chinchón (FC), and Violeta Spring (VS). Except for FC, which is locally restricted to the south of Madrid, these cultivars are among the main varieties in Spanish garlic production [52]. FC is a traditional and appreciated cultivar from the Chinchón area [59]. MP is recognized by the European Union as a Protected Geographical Indication or PGI [60]. GA is an introduced “American type” commercial variety, whose cultivation is currently widespread in Spain. VS is an introduced “Chinese type” cultivar, which matures early unlike the other varieties [53]. These 4 cultivars were selected due to the widespread and economic importance of their cultivation and because they cover a wide range of variability with respect to their origin and characteristics. According to the classification by Lallemand et al. [61], MP, FC, GA, and VS are included in the groups I, II, III, and IV, respectively [53].

For garlic germination, cloves from each bulb were individually separated, immersed in tap water, and kept at 4 °C for 24 h. If a garlic clove did not germinate, it was maintained in water for a longer period (48 h), or moved to a Ziploc plastic bag to conserve moisture. Germinated garlic cloves were planted in pots filled with a mixture of river sand and organic substrate in a 2:1 ratio, previously autoclaved for 20 min at 118 °C. In most cases, potted plants were moved to a growth chamber to carry out the assays under controlled conditions involving a temperature and light regime. Moreover, a few assays were repeated in a shade house to test conditions more similar to those of natural cultivation in a field.

### 4.2. Initial Conditions for the Growth Chamber Assays

Germinated garlic, individually planted in 15 cm diameter pots, were moved to a growth chamber with an L9:D15 h photoperiod and L12:D6 °C temperature, to simulate the initial conditions for growing garlic in open field cultivation. Over time, the temperatures and light period were progressively increased, in order to simulate the natural evolution of environmental conditions as much as possible.

### 4.3. Initial Conditions for the Shade House Assays

The shade house was 6 × 3 × 2 m in size, surrounded by an anti-trip net and double shaded when needed to avoid excessive temperature. In this case, four garlic cloves were placed together in a large pot with a diameter of 35 cm and a height of 50 cm. Each pot was placed on a tray, which in turn was placed on top of a plastic grid to avoid excessive heating of the soil from the bottom. These assays began in December, at the same time that garlic crops are usually started in a field, with light and temperature conditions evolving similarly.

### 4.4. Water Treatments

The soil moisture in each pot, expressed as %VWC (volumetric water content), was monitored daily at the beginning of each assay (and every 2–4 days thereafter) by means of a ProCheck soil moisture logger with a sensor ECH2O EC-5 and Teros-10 (Decagon Devices^®^, Inc., Pullman WA, USA). In the first phase of each assay, all the plants were watered abundantly to facilitate their growth. After a few days, all the plants continued to be watered similarly, but only when the soil moisture was ≤25% VWC. This irrigation regime was considered as the control (C) treatment because, in previous studies with garlic, it was observed that the closure of the stomata starts at ≤20% VWC, approximately [36]. At 2–4 weeks after germination, half of the plants continued in the control conditions and the other half were subjected to a lower irrigation regime, being watered only when the soil moisture was ≤12% VWC. This was considered as the water stress (S) treatment, and the difference with the C treatment was maintained until the end of the assay. A minimum of 10 plants per water treatment were considered, and each assay was repeated at least twice.

**Figure 5:**
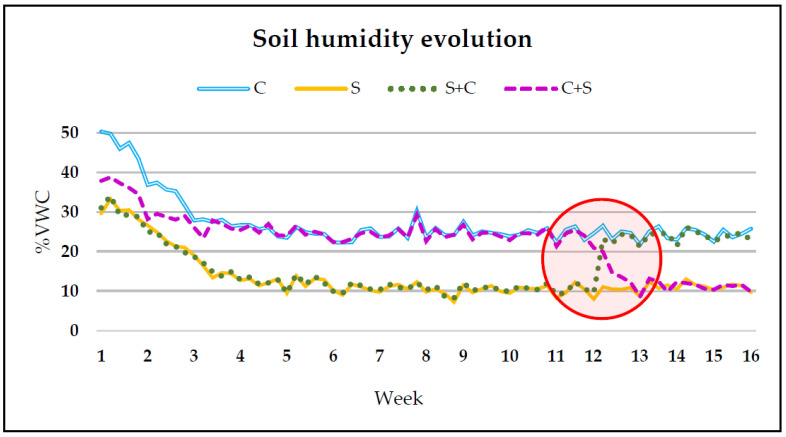
Evolution of soil moisture data from garlic transplanting until harvesting. The timing of the change from the control (C) treatment to stress (S) and vice versa is highlighted inside the circle.

In other assays designed to observe the influence of continuity or discontinuity of the water stress, half of the control plants were switched to the stress treatment (C+S) at one point in time, and half of the stress plants were returned to the control regime (S+C). An example of the evolution in the moisture data with the different treatments is shown in Figure 5.

### 4.5. D. dipsaci Infestation

Nematodes for plant inoculation were obtained from garlic bulbs naturally infested with *D. dipsaci* in the field. Garlic cloves were separated from the bulbs, placed on a sieve with a 250 µm pore diameter, immersed in tap water in a plastic bowl, and maintained at 4 °C for 24–48 h to facilitate nematode extraction. After this time, the volume of the liquid containing the nematodes was reduced by filtering through a 25 µm sieve to retain the nematodes. The sieve with the retained nematodes was carefully washed to collect the nematodes in a beaker, recording the total volume. This extraction method is a modification of that by Nombela and Bello [62].

A stereo microscope and a Malassez counting chamber were used to identify and count the nematodes for plant inoculation. Only the adults and fourth stage juveniles (J4) of *D. dipsaci* contained in an aliquot of 500 μL were counted and the mean value obtained from 3 aliquots was extrapolated for the total volume of the sample.

**Figure 6:**
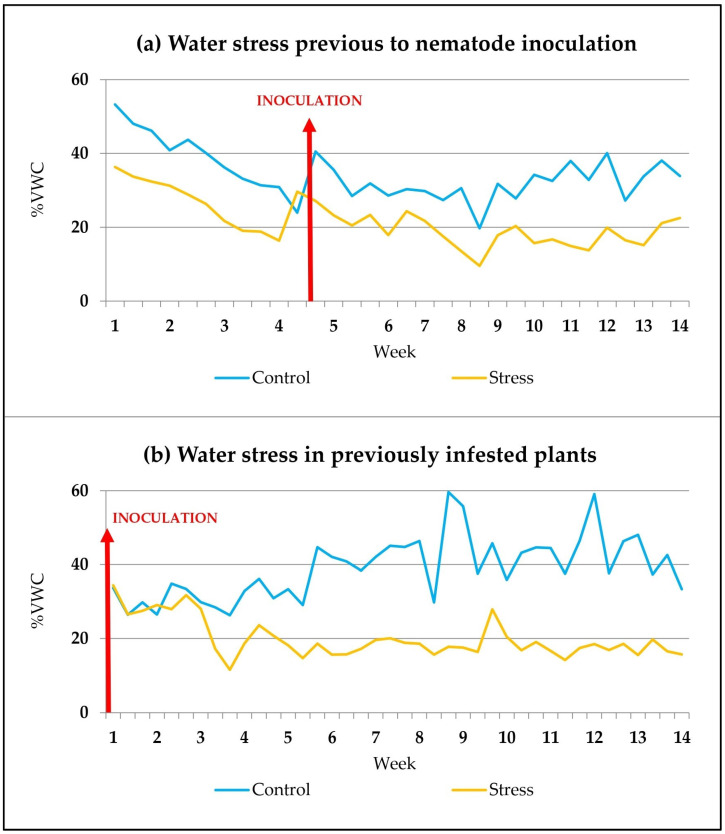
Evolution of soil moisture data when water stress treatment was applied before (**a**) or after (**b**) nematode inoculation (red arrow). Each data is the mean value of the moisture measurements for all plants with the same water treatment.

Approximately 5 weeks after the start of the water stress treatment (Figure 6a), each stressed or control garlic plant was inoculated with 6000–7000 nematodes, depending on the assay. The suspension containing the nematodes was deposited by means of a pipette at the base of the plant stem and the closest soil.

In other assays, naturally infested plants were used, or nematodes were inoculated only 10 weeks after the germination of normally watered plants. The start of the water stress treatment in half of these previously infested plants was carried out 2 weeks after nematode inoculation (Figure 6b).

### 4.6. Nematode Extraction and Counting

**Figure 7:**
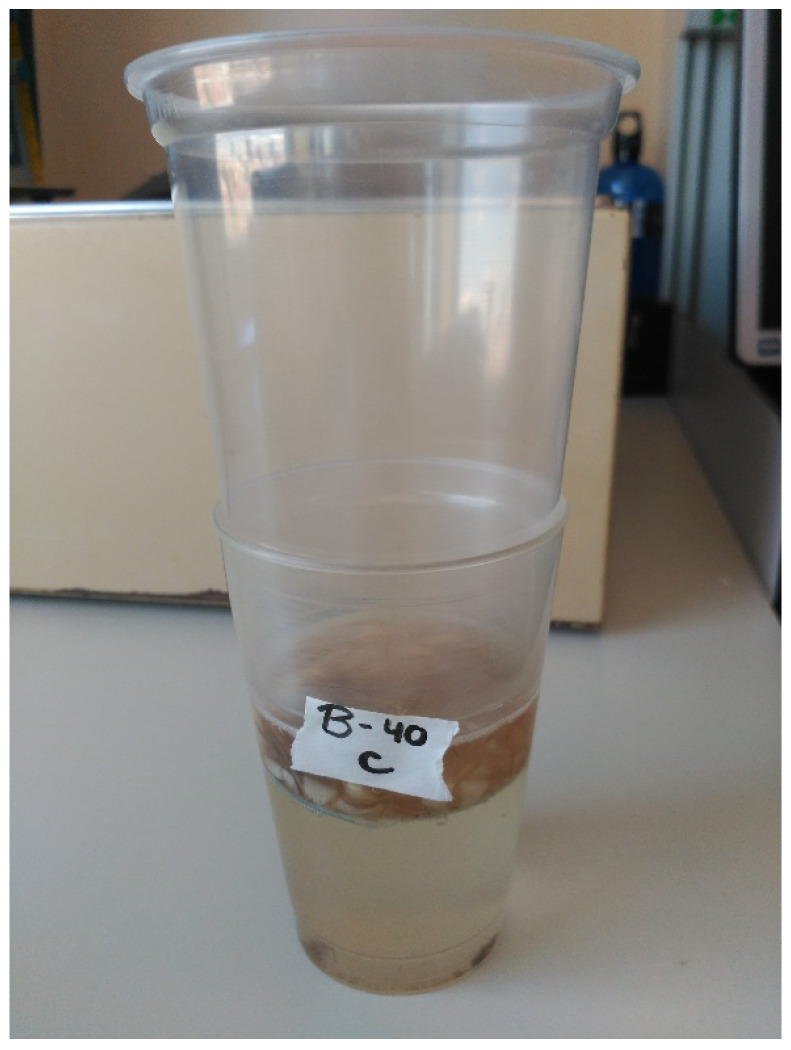
Plastic cup system for nematode filtration with 250 µm mesh nylon filter.

At least eight weeks after inoculation, the aerial part of each plant was cut (preserving one centimeter of the stem base, where some nematodes could also be found), immediately frozen and stored at −80 °C until the determination of the physiological parameters was carried out. The bulb and roots were carefully taken out of the pot, removing as many adhering soil particles as possible. They were lightly washed, dried with filter paper, and weighed. Subsequently, the bulb and roots were cut with a scalpel into very small pieces, which were deposited in a plastic cup with the bottom replaced by a 250 µm mesh nylon filter. This filter was placed inside another plastic cup filled with enough water to cover all the bulb pieces (Figure 7). This filtration system, kept for 48 h at 4 °C temperature, retained the garlic pieces, but allowed the nematodes to pass through the filter into the second beaker. After this time, the adults and J4 of *D. dipsaci* were counted in each sample using a stereo microscope, as described above, to obtain the number of nematodes per plant.

### 4.7. Plant Physiology Parameters

Garlic extracts were obtained from the aerial part of the nematode-infested or non-infested plants that had been separated from their bulbs and preserved at −80 °C. In all cases, sample triplicates were analyzed and the experiment was repeated twice.

#### 4.7.1. Total Chlorophyll Determination

Garlic extracts were obtained from 50 mg of leaf tissue, and the simultaneous quantification of chlorophyll a and b were spectrophotometrically determined, following Arnon’s methods [63].

#### 4.7.2. Proline Concentration

From garlic leaves, 100 mg of tissues were used to prepare and extract proline using 3% of sulfonic acid. Following the incubation with ninhidrine at 90 °C and separation with toluene, the L-proline concentration was determined spectrophotometrically at 540 nm [64].

### 4.8. Statistical Analysis

Statistical analyses were performed using IBM SPSS Statistics 27.0.1, Armonk, NY, USA software for the parameters considered (nematodes/plant and bulb and root weight). The mean and standard error values were obtained for each water treatment and garlic variety. Each data series were subjected to normality analysis (Shapiro–Wilk test), homogeneity of variance (Levene’s statistical test), and the outlier data were discarded. Data adjusted to a normal distribution were analyzed by a one-way ANOVA, and the means were compared by the Tukey’s HSD post hoc test to determine which treatments were significantly different. When the data were not adjusted to a normal distribution, they were log10(x + 1) transformed before analysis. When the data, after transformation, were still not adjusted to a normal distribution, the means were compared using the Mann–Whitney U test for 2 treatments or by the Kruskal–Wallis test for more than 2 treatments. The statistical significance of the differences in the chlorophyll content and proline concentration data were checked using the Kruskal–Wallis test.

## 5. Conclusions

This study has revealed for the first time that drought has a notable impact on the host response by four garlic cultivars to a *D. dipsaci* attack, as a consequence of the changes produced in certain parameters related to plant growth (bulb and root weight) and metabolism (chlorophyll and proline content). In addition, the obtained results allow us to conclude that the time at which the plant is subjected to water stress in relation to the infection by the nematode is a determining factor in the host response of garlic. So, a pre-existing reduced water supply resulted in low levels of infection by *D. dipsaci*, meanwhile drought affecting previously infected plants caused an increase in the final nematode populations. In cases where water stress was discontinuous, the results exhibited marked inter-cultivar variability.

The results from the present work lead us to recommend that garlic cultivation in arid areas should preferably follow the traditional rainfed system, which will help to reduce the risk of yield losses due to plant-parasitic nematodes, such as *D. dipsaci*. In the present scenario, under water scarcity circumstances, with emerging pests and pathogens, and with crop genetic variability decreasing due to the substitution of local and traditional cultivars by commercial, more economically profitable varieties, it will be necessary to select garlic genotypes that exhibit higher resistance or tolerance, not only to drought but also to nematodes and other plant-pathogenic organisms.

## Figures and Tables

**Table 1:** Number of *D. dipsaci* per plant (mean ± standard error) obtained at the end of the assays from plants subjected to a reduced irrigation regime starting **4 weeks before nematode inoculation** (stress), or never subjected to water stress (control).

Cultivar *	Nematodes/Plant		F or U **	*p*
	Control	Stress		
GA	6176.15 ± 920.66 a	616.46 ± 151.28 b	U = 0.000	<0.01
MP	2510.00 ± 385.85 a	166.80 ± 108.67 b	U = 1.000	<0.001
FC	18,060.42 ± 5111.20 a	8800.00 ± 4206.20 b	U = 31.000	0.032
VS	3592.47 ± 276.16 a	2051.10 ± 165.19 b	F = 20.485	<0.001

* GA: Gardacho, MP: Morado de las Pedroñeras, FC: Fino de Chinchón, VS: Violeta Spring. ** For each garlic cultivar, different letters represent significant differences (*p* < 0.05) in the number of nematodes between the control and stress water treatments, compared using a one-way ANOVA (F value) or using the Mann–Whitney test (U value).

**Table 2:** Number of *D. dipsaci* per plant (mean ± standard error) obtained at the end of the assays from plants subjected to a reduced irrigation regime initiated **at least 2 weeks after nematode inoculation** (stress), or never subjected to water stress (control).

Cultivar *	Nematodes/Plant		F or U **	*p*
	Control	Stress		
GA	2186.63 ± 291.71 a	2635.56 ± 450.11 a	F = 0.662	0.958
MP	1913.33 ± 302.75 a	3937.08 ± 744.72 b	U = 38.000	0.520
FC	5722.25 ± 789.53 a	5642.67 ± 1090.45 a	F = 0.003	0.954
VS	75,995.96 ± 15,452.28 a	90,303.96 ± 20,533.42 a	F = 0.322	0.584

* GA: Gardacho, MP: Morado de las Pedroñeras, FC: Fino de Chinchón, VS: Violeta Spring. ** For each garlic cultivar, different letters represent significant differences (*p* < 0.05) in the number of nematodes between the control and stress water treatments, compared using a one-way ANOVA (F value) or using the Mann–Whitney test (U value).

## Data Availability

Data are contained within the article or are available on request from the corresponding author.
